# How do staff and team characteristics relate to ward safety incidents in adult inpatient mental health settings? A protocol for a systematic integrative review

**DOI:** 10.1136/bmjopen-2025-110675

**Published:** 2026-03-11

**Authors:** Katy Greenfield, Bethany Griffin, Sarah Kendal, Samuel Woodnutt, Nutmeg Hallett, Judith Johnson, Kathryn Berzins, Chris Bojke, Max Henderson, Jo Lomani, Emma Wadey, John Baker

**Affiliations:** 1 School of Healthcare, University of Leeds, Leeds, UK; 2 School of Health Sciences, University of Southampton, Southampton, UK; 3 School of Nursing and Midwifery, University of Birmingham, Birmingham, UK; 4 Division of Nursing, Midwifery and Social Work, The University of Manchester, Manchester, UK; 5 Health Technology Assessment Unit, Applied Health Research Hub, University of Central Lancashire, Preston, UK; 6 Academic Unit of Health Economics, University of Leeds, Leeds, UK; 7 Leeds Institute of Health Sciences, University of Leeds, Leeds, UK; 8 NHS England North, Leeds, UK; 9 Hertfordshire Partnership University NHS Foundation Trust, Hatfield, UK

**Keywords:** MENTAL HEALTH, Safety, Inpatients, Nursing research, Review

## Abstract

**Abstract:**

**Introduction:**

A neglected area of patient safety research is how the characteristics of mental health staff and teams may influence incidents, specifically, through unintended and harmful consequences of clinical care. While the research literature into patient safety has increased, there is still a need to further consider safety on mental health wards, for example, the role of the staff team in containment and conflict. This review aims to explore the question, ‘How do staff and team characteristics relate to safety incidents in adult inpatient mental health settings?’.

**Methods and analysis:**

The review will follow Whittemore and Knafl’s integrative review framework. CINAHL, Cochrane, Embase, MEDLINE, PsycINFO, Web of Science will be searched. Literature published after 1999, that includes extractable quantitative, qualitative and mixed methods data exploring the relationship between staff and team characteristics on incidents in adult inpatient mental health settings, will be suitable for inclusion. The Mixed Methods Appraisal Tool will be used for quality appraisal and data analysis and will comprise data reduction, display and comparison.

**Ethics and dissemination:**

No new data or access to participants will be involved in this review. As such, ethical review will not be required. Dissemination will include publication in peer-reviewed journals and presentations at national and international conferences.

**PROSPERO registration number:**

This review has been registered on PROSPERO (ref. CRD420251119981; https://www.crd.york.ac.uk/PROSPERO/view/CRD420251119981).

**Strengths and limitations of this study**

- The proposed methodology has been reviewed by a team of experts in the field, including lived experience experts and an information specialist.
- Independent reviewers and a third-party reference for disagreements during screening, data selection and appraisal enhance the robustness of the proposed review.
- The search will be limited to English language only abstracts to ensure feasibility within the teams’ resources.

## Background

 Patients on mental health wards should be safe; yet medical and adverse events (unintended and harmful consequences of clinical care) are common.^1^ Around one in ten acute mental health hospital admissions involves a medical error, and one in six an adverse event.^1 2^ Alongside risks faced by general healthcare hospital patients, for example, errors or missed/omitted care due to staffing shortages,^3^ mental health inpatients face particular risks such as aggression, self-harm, absconding and suicide.^4^ Mental health staff typically respond and try to prevent harm to patients with restrictive interventions such as restraint and seclusion,^2^ which have a noted detrimental impact on patients.^5^ Yet despite expressed concerns,^6 7^ patient safety in mental health wards is poorly understood and under-researched.^2^ An especially neglected area is how the characteristics of mental health staff and teams may influence safety.^6 8 9^ There have been suggestions for relevant factors to be considered in relation to safety on mental health wards, for example, the role of the staff team in containment and conflict.^10^

Previous reviews have demonstrated links between staff characteristics and a range of outcomes in inpatient mental health settings, but the breadth of staff characteristics and outcomes studied has been restricted and varied widely, failing to provide a holistic overview. For example, staff characteristics such as age, gender, ethnicity, role and experience may influence the use of restrictive interventions^11^ or the reporting of incidents.^12^ Research on patient safety priorities highlights the importance of staff competence and listening skills.^11^ Staff attitudes, values and beliefs may also be relevant in relation to the implementation of restraint minimisation interventions.^13^ Staffing attitude, personal history and staffing levels may also be associated with the noticing of, and therefore the reporting of safety incidents.^12 14^ Safety incidents in these reviews are often decided deductively and thus limited to aggression, absconding and self-harm and the measures used to contain these, such as the use of seclusion and restraint. Synthesising the literature across a range of staff and team characteristics, and their impact on a variety of safety incidents is therefore needed to progress safety research in inpatient mental health.

The mental health context is unique^2^: patients have described how the unpredictability of mental health wards may have traumatised them at their most vulnerable,^9^ potentially extending the length of hospital admission. While there is a growing body of evidence exploring patient safety in mental healthcare, empirical evidence is still lacking compared with general healthcare settings.^2^ In addition, mental health staff suffer higher levels of burnout than other healthcare staff, with more sickness absence days and work-related stress.^8^ This can cause them to become emotionally distanced, in turn leading to negative attitudes towards patients and higher risk of incidents.^8^ Negative staff attitudes and staff making errors have both been linked with increased patient violence.^15^ There is a specific lack of evidence regarding how staff and team characteristics may impact incidents. Utilising systematic integrative review methodology will enable this wide literature to be searched and collated, not limited by study design.

At present, there are no theoretical models of the interaction between incidents on mental health wards and staffing characteristics. However, factors of the Yorkshire Contributory Factors Framework adapted for mental health services (YCFF-MH)^6^ can be considered for safety incidents. The YCFF-MH is an adapted framework building on the YCFF, a framework developed through a systematic review to understand factors contributing to patient safety incidents in hospital settings,^16^ that expanded the scope to include patient safety factors in mental health settings. The five relevant factors within the framework include: active failures, situational failures, local working conditions, latent organisational factors and latent external factors. Findings from the current review will provide new evidence to expand and refine theoretical models such as the YCFF-MH, specifically the consideration of staff and team characteristics as a contributory factor.

### Aim

This review aims to explore the question, ‘How do staff and team characteristics relate to ward safety incidents in adult inpatient mental health settings?’.

## Methods

The review will follow Whittemore and Knafl’s integrative review framework.^17^ The five stages of this methodological framework are: (1) problem identification, (2) literature search, (3) data evaluation, (4) data analysis and (5) presentation. A systematic integrative review approach was selected because it enables the synthesis of both qualitative and quantitative literature and thereby generates both the ‘what’ (which factors are relevant) and the ‘why’ (potential causal mechanisms and underlying associations). This protocol was reported in line with the PRISMA-Protocols (PRISMA-P) checklist (included in the online supplemental material files S1). Design and conceptualisation started 21 July 2025 and the review is expected to be completed by April 2026.

### Patient and public involvement

This review was conceptualised in consultation with people who have lived experience of inpatient mental healthcare. Experience experts contributed to the development of the review protocol. The wider research team includes a Lived Experience Expert who facilitates the project’s dedicated Lived Experience Advisory Group (LEAG). The LEAG advises on a wide range of research decisions and will be involved in the interpretation and dissemination of findings from the review.

### Condition or domain being studied

The focus of this review will be on exploring staff and team characteristics and their relationship to safety incidents that occur in adult inpatient mental health wards. The following definitions have been developed, based on previous literature and collaboration with experts in the field, including people with lived experience of inpatient mental health settings. Staff characteristics refer to anything personal or professional within and about the individual staff member providing care, for example gender, ethnicity, personality and experience. We use the term ‘team’ in this review to mean all of the people who together provide support to patients in an adult inpatient mental health setting. Thus, team characteristics refer to the interaction of qualities and skills of the people providing care. A safety incident refers to something unexpected or unintended that has happened or failed to happen, that could have or did lead to harm. This definition includes harm to anyone on the ward, including staff.

### Eligibility criteria

We will conduct systematic searches of academic databases to identify literature published after 1999 that reports on staff and team characteristics and their relation to safety incidents in adult mental health inpatient settings.

#### Inclusion criteria

- Published original articles that include extractable quantitative, qualitative and mixed methods (including case studies) data exploring the relationship between staff and team characteristics on incidents. A mixed-methods approach will enable a comprehensive review of the current literature, combining both observational and experimental studies to understand the impact of staff and team characteristics on safety incidents.
- Published literature including staff, patients, and families and carers of patients based in adult inpatient mental health settings.
- Literature published after 1999, that is, since the first UK National Service Framework of quality standards for mental health services and the emerging concept of ‘patient safety’.^18^
- Literature in any language that includes a readily available English abstract

#### Exclusion criteria

- Reviews, editorials and book chapters will be excluded at screening stage, as it is unlikely that these sources will yield new material. Their reference lists will be searched for relevance.
- Literature solely reporting on the experience of people receiving care or working in Children and Young People’s Mental Health Services, outpatient services, dementia wards or older adult inpatient mental health wards.
- Literature reporting on inpatient mental health settings offering services solely to people under 18 years old and over 65 years of age.
- Literature in languages other than English that does not have an English language abstract readily available.

### Information sources

The following databases will be used as part of the search strategy for published studies: CINAHL [EBSCOhost], Cochrane, Embase [Ovid<1996 to 2025 Week 36>], MEDLINE [Ovid<1946 to September 08, 2025>], APA PsycINFO [Ovid<1806 to September 2025 Week 1>], Web of Science. These databases are best suited to provide literature pertinent to the review aims. PsycINFO, Medline and CINAHL cover literature on healthcare workforce and services of interest, while Web of Science covers both medical and social sciences to provide information on the professional context. The addition of Embase covers a broader international perspective and Cochrane will provide reviews that may be relevant for hand searching. MeSH subject headings and abstract and title keyword searches will be used per each database. The reference lists of relevant papers will also be searched.

### Search strategy

Search strings will be constructed using three key concepts/blocks: workforce, setting (inpatient mental health setting) and incident to operationalise the phenomenon of interest. After individual pilot database searching in August 2025 and subsequent team discussion, a decision was made not to include ‘characteristics’ within the search terms, to ensure a broad net was cast and reduce the possibility of potentially pertinent papers being excluded. This will be an inductive review, to discover what, and how, staff characteristics may be involved in safety incidents on mental health wards, and as such, we would be limiting our search by our current understanding of what they might be, potentially missing papers which could provide further insight. Consideration will also be given to the international context of the review and include terms used in other countries for workforce, setting and incident descriptors. The full search strategy is available through the host organisation’s repository.^19^

### Selection of sources of evidence

Results from each database will be exported into the reference manager software Rayyan where duplicate entries will be removed, using Rayyan’s automation tool and manually checked by KG. Study selection will consist of two stages, with the first involving the screening of titles and abstracts. This will be shared between two reviewers (KG and BG) independently screening 50% of the titles and abstracts each. Each reviewer will screen 10% of the other reviewers’ share to check agreement. Reviewers will meet following initial screening in which disagreements will be discussed and resolved. If an agreement is not reached, the opinion of a third independent reviewer will be sought (SK). In the second stage, full text screening will be carried out independently by KG and BG and checked with the research team to ensure agreement. Each of these stages and the processes involved will be consolidated into a PRISMA flow diagram.

### Data charting process

Data charting will be shared between BG and KG. SK will independently extract data from 10% of papers to ensure agreement. Data will be charted into a Microsoft Excel spreadsheet but will follow an inductive approach (involving exploring the data to understand patterns), as outlined in guidance for integrative methodologies.^17^ Charted variables for each article may include, but are not limited to: author, year, country of data collection, sample profile, study design, staff characteristics reported, team characteristics reported, ward safety incident outcome and key findings.

### Quality appraisal

The Mixed Methods Appraisal Tool will be used for quality appraisal of the included academic literature.^20^ The tool is suitable for the appraisal of diverse literature, including the critical appraisal of quantitative, qualitative and mixed-methods studies. While poorer quality papers will not be removed from the synthesis, the quality of each study will be mapped to the analysis stage for reference. The quality of the research included for review could impact the validity of the findings. As such, exploring and reporting the quality of included papers allows for reflection and recommendations for conducting future research in this area.

### Strategy for data synthesis

Our approach to data synthesis is informed by our knowledge of the literature in this field, which is large and heterogeneous. To clarify whether a meta-analysis would be feasible, we conducted a preliminary scoping search of Ovid Medline from inception to 1 May 2024. Three blocks of search terms relating to: Adult mental health wards/hospital AND Staff and team characteristics AND Patient safety were used. This search drew 53 hits, of which only two would be eligible for a meta-analysis examining associations between staff/team factors and incidents in mental health wards. Furthermore, it would not be possible to synthesise these two studies within the same analysis, due to heterogeneity in the staff/team and incident outcome factors studied. Preliminary searching indicates that in addition to being poorly suited to our research question, a meta-analysis of available data is unlikely to be feasible, especially given the range of methodologies which will be included in the review. Therefore, data analysis will comprise data reduction, display and comparison.^17^

## Discussion

Previous research suggests that the most common safety incidents could be nursing-sensitive,^14^ that is, resulting from nursing care; however, these findings are reflected in a limited body of mental health research, and the overall picture is unclear. Safety incidents cause considerable short and long-term harm to patients and staff and substantially increase organisational costs. Despite extensive literature on violence and restrictive interventions, evidence for the impact of mental health staff and team characteristics on patient safety is mostly absent, especially when compared with other hospital settings. If factors that increase the risk of safety incidents can be recognised, then interventions that target those factors could reduce risks.

The development of the proposed protocol has been reviewed by a team of experts in the field, including lived experience experts and an information specialist, which has helped to refine the scope and strengthen methodological rigour. Including independent reviewers and a third-party reference for disagreements during the screening, data selection and appraisal process further enhances the robustness of the proposed review. It is recognised that limiting the search to include English language only abstracts is a limitation to ensure the study is feasible within our resources. To mitigate the effects of this, we will include papers in other languages that have published an English language abstract to be considered for translation and potential inclusion.

The scope of this review will be broad to include a range of individual staff and team characteristics and safety incident outcomes. The results of our wide-ranging integrative review will add knowledge by generating new insights to inform policy and practice worldwide. They can be translated into realistic recommendations for organisations to consider and incorporated in a practical way to inform operational practice and clinical education and training. We anticipate, therefore, that the review findings will make a significant contribution towards improved safety in inpatient mental health settings.

### Project status

The current protocol was originally submitted for peer review in September 2025, prior to formal searching. As of January 2026, data extraction is underway.

## Supplementary material

10.1136/bmjopen-2025-110675 online supplemental file 1
