# Supplementary material for: How do staff and team characteristics relate to ward safety incidents in adult inpatient mental health settings? A protocol for a systematic integrative review
Source: BMJ Open. 2026 Mar 11;16(3):e110675. doi: 10.1136/bmjopen-2025-110675 (PMC12983825; doi:10.1136/bmjopen-2025-110675)
Supplement: Supplementary file 2 [file bmjopen-2025-110675.reviewer_comments.pdf]

PEER REVIEW HISTORY

BMJ Open publishes all reviews undertaken for accepted manuscripts. Reviewers are asked to complete a checklist review form and are provided with free text boxes to elaborate on their assessment. These free text comments are reproduced below.

ARTICLE DETAILS

Title (Provisional)

How do staff and team characteristics relate to ward safety incidents in adult inpatient mental health settings? A protocol for a systematic integrative review.

Authors

Greenfield, Katy; Griffin, Bethany; Kendal, Sarah; Woodnutt, Samuel; Hallett, Nutmeg; Johnson, Judith; Berzins, Kathryn; Bojke, Chris; Henderson, Max; Lomani, Jo; Wadey, Emma; Baker, John

---

VERSION 1 - REVIEW

**Reviewer**	**1**
**Name**	**Chakraborty, Nandini**
**Affiliation**	**Leicestershire Partnership NHS Trust**
**Date**	**27-Dec-2025**
**COI**	**None**

The abstract should mention the timelines of the studies to be included in this review.

'To protect against the exclusion of relevant material there will be no

restrictions by study design and quality. As such, this includes original articles,

reviews, editorials, book chapters and case studies'.

I wonder if this would produce any new material. Since reviews, editorials and book chapters will be based on already published material/references, these sources are unlikely to produce first hand information. They will be opinions of the authors based on references which the current authors might be better to include in their list of primary references for this review.

I would suggest excluding reviews, editorials, book chapters and case studies as such but they might be perused by some of the authors in the current review team to see whether they yield references to original studies which might be included in the review.

I could not see a specific definition of 'safety incidents'. There is a mention of a range starting from medication errors to absconding, self-harm and aggression. There is no specific

mention of suicide, though this might be an incident with deep ongoing impact on both patients and staff. There might be studies which have looked at incidents widely or specific types of incidents. The search terms do include an extensive array of terms in including both suicide and self-harm. However, the mention of suicide in the text amongst the types of incidents being looked at is appropriate.

Looking at the search terms the characteristics of the workforce has been explored in terms of the kind of professionals in the team. Foreign medical graduates have been mentioned. I think foreign graduates or better termed overseas graduates for all disciplines is significant, not just medical graduates.

The other aspect in the composition of the workforce which would be notable to explore is permanent versus locum/bank staff. Does this impact on incidents would be significant. Bank/locum, permanent staff could be factored into the search words for workforce.

It is an important area of study and as the authors put forward, literature is limited. The review findings should be interesting and have an impact on workforce planning on dissemination.

**Reviewer**	**2**
**Name**	**Deering, Kris**
**Affiliation**	**University of Exeter, Academy of Nursing**
**Date**	**08-Jan-2026**
**COI**	**None**

Dear authors.

Thank you for considering BMJ open for this very interesting systematic integrative review protocol. I appreciate the work involved in making the paper ready for peer review.

In my view the topic is very suitable for the journal and of an important subject. Exploring clinical staff/team characteristics within mental health hospitals and how these influence incidents of safety on wards is certainly an underexplored topic.

On the whole, I found the paper very well written, informative and accessible. In spirit of developing the paper, I have made suggestions below about how areas could be edited. By all means if you disagree, please note these disagreements in the response letter.

Abstract.

Introduction.

Line 8 Page 3: I was left wondering somewhat how safety incidents were contextualised as well as the clarity of the introduction section. Early on in the paper safety incidents is more defined by stating ‘unintended and harmful consequences of clinical care’. Would it be

possible please to use this ‘definition’ in the introduction? For example (I changed some wording which in my view aided clarity).

A neglected area of patient safety research is how the characteristics of mental health staff and teams may influence incidents, specifically, through unintended and harmful consequences of clinical care. Despite this, there is need to consider safety on mental health wards, for example the role of the staff team in containment and conflict. This review aims to explore the question, “How do staff and team characteristics relate to incidents in adult inpatient mental health settings?”

Background.

Line 31 Page 4: There have been suggestions for relevant factors to consider in relation to safety on mental health wards, for example the role of the staff team in containment and conflict (10). I think ‘to be considered’ was meant to keep in the past tense?

Line 45 Page 4: Perhaps add the term safety to ‘incidents (12)’.

Line 3 Page 5: I wonder if this sentence could be slightly adapted to ‘Synthesising the literature across a range of staff and team characteristics, and their impact on a variety of safety incidents is therefore needed to progress safety research within inpatient mental health care’.

Line 13 page 5. In no way do I recommend the referencing of the following paper, especially given that I was one of the authors. But I would like to point out that there have been recent developments in relational care to aid patient feeling safe (in this case with their recovery) within acute inpatient settings. This, in turn makes me wonder a little about the accuracy of the following point: - ‘There appears to be a lack of empirical evidence from mental health wards exploring patient safety in these settings compared to general healthcare settings.’ If you wish to know more please see Deering, K., Wagstaff, C., Kyle, R.G., Bermingham, I., Williams, J. and Pawson, C., 2025. Safety for Recovery During Admission to Acute Mental Health Hospitals: A Constructivist Grounded Theory Study to Support Safety-Focused Recovery-Oriented Care in the Context of Risk Management Constraints. International Journal of Mental Health Nursing, 34(4), p.e70104. – thank you.

Line 23 Page 5: Perhaps change staff error to staff errors or staff making errors? This is so it is clear that this refers to staff actions, not for example error in skills mix e.g. staffing error. Though of course this could be an issue.

Line 28 Page 5. Perhaps a little pentadic on my part but ‘allow’ can regard permission while enable is for more causal to the methodological approach. E.g. will enable a wide range…

Aim

Line 56 page 5. The question is slightly different as missing the term safety e.g. safety incidents. If OK with you when referring to the question just use the title of the paper - How do staff and team characteristics relate to ward safety incidents in adult inpatient mental

health settings? Also, I was left wondering if the focus was on acute inpatient settings or all types of mental health inpatient settings. It might help therefore to clarify this also in the aim. This also could mean adapting to title of the paper to include acute, if seen reasonable to do so.

Eligibility

I appreciate in the research terms at the end of the paper you are looking at particular dates but this is not clear in this section. Please add date range and rationale for searching the papers. Apologies if this is in this section but I could not see it. I also wonder if there might be a rationale provided for a mixed studies approach to the review? So, for example to develop a comprehensive picture around the impact of staff/team characteristics through observational and/or experiential studies.

Line 57 page 6: Regarding 'Literature including staff, patients, and families and carers of patients based in adult inpatient mental health settings', is this literature published also?

Information sources

Line 39 Page 7. It might be beneficial to provide a brief rationale for searching the identified databases in terms of the aim of the review.

Search strategy

Line 52 Page 7: I wonder if the search should include the search term "characteristics" and another search without, as without investigating the term, this too might result in overlooking papers? Moreover, as I understand it, the term was utilised to understand the usefulness of conducting a meta-analysis later in the paper. This might leave readers wondering why the search strategy mentioned here was not utilised for understanding the need for a meta-synthesis approach.

Selection of sources of evidence

Line 10 page 8. I found this paragraph using a few incorrect terms and syntax. Please see a proposed edit of the paragraph as guidance. The changes are highlighted using [ ].

Results from each database will be exported into the [reference manager] software Rayyan where duplicate entries will be removed. Study selection will consist of two stages with the first [involving the] screening of titles and abstracts. This will be shared between two reviewers (KG and BG) independently screening 50% of the titles and abstracts each. Each reviewer will screen 10% of the other reviewers share to check agreement. Reviewers will meet [following] initial screening [deleted] [in which] disagreements will be discussed and resolved. If an agreement is not reached, the opinion of a third independent reviewer will be sought (SK). In the second stage, full text screening will be carried out independently by KG and BG and checked with the research team to ensure agreement. Each of these stages and the processes involved will be [consolidated into] a PRISMA flow diagram.

Data charting process

Line 36 Page 8: Following inductive approach it might help the readers to understand its meaning, I suggest the following …will follow an inductive approach (involving exploring the data to understand patterns), as outlined in guidance for integrative…

Quality appraisal

Line 52 Page 8: I am wondering if there should also be a mention about how exploring the quality of research can impact on the validity/trustworthiness etc of findings in review, and that such analysis will inform recommendations in the review to inform the conducting of future research on the topic.

Strategy for data synthesis

Line 5 Page 9: Perhaps write - which is large and [not but] heterogeneous.

Line 20 Page 9: I wonder if a meta-analysis was also not prudent due to different quantative study designs making it difficult to expand a sample size?

Discussion

Line 32 Page 9: Regarding, 'short- and long-term', please remove the dash after short.

Line 55 Page 9 I think 'we have imposed' could be removed.

Line 57 Page 9: A comma is needed after 'To mitigate the effects of this'.

Line 14 Page 10: Regarding, 'in a practical way into operational…' I think in a practical way to inform operational – is perhaps better.

Line 16 Page 10: Regarding, 'We anticipate this can make a significant contribution towards improved safety in inpatient mental health settings'. Again, I might be being a bit pedantic here but 'this' was not too clear to me. Using [ ], I wonder if the following is useful. We anticipate[, therefore, that the review findings will] make a significant contribution towards improved safety in inpatient mental health settings.

Thank you for the opportunity to review this interesting paper and I wish you the very best with having it published.

VERSION 1 - AUTHOR RESPONSE

Reviewer 1	Response
The abstract should mention the timelines of the studies to be included in this review.	The inclusion criterion of literature published after 1999 has now been added to the abstract.
'To protect against the exclusion of relevant material there will be no restrictions by study design and quality. As such, this includes original articles, reviews, editorials, book chapters and case studies'.	Thank you to the reviewer for their reflections on this. Since initial submission to BMJ Open, we have now completed searching and screening and have since made the decision to exclude reviews, editorials and book chapters as

I wonder if this would produce any new material. Since reviews, editorials and book chapters will be based on already published material/references, these sources are unlikely to produce first hand information. They will be opinions of the authors based on references which the current authors might be better to include in their list of primary references for this review.<br>I would suggest excluding reviews, editorials, book chapters and case studies as such but they might be perused by some of the authors in the current review team to see whether they yield references to original studies which might be included in the review.	primary references and have instead hand searched these for relevant references. A sentence clarifying this has been added to bullet point one of the exclusion criteria (page 6).
I could not see a specific definition of 'safety incidents'. There is a mention of a range starting from medication errors to absconding, self-harm and aggression. There is no specific mention of suicide, though this might be an incident with deep ongoing impact on both patients and staff. There might be studies which have looked at incidents widely or specific types of incidents. The search terms do include an extensive array of terms in including both suicide and self-harm. However, the mention of suicide in the text amongst the types of incidents being looked at is appropriate.	Suicide as a risk that patients face in inpatient mental health has been added to the background (page 3, paragraph 2). A definition of incidents was included on page 5 (condition or domain being studied) but did not specifically mention 'safety incidents'. For consistency of language, we have now rectified this to 'safety incident' throughout the manuscript.
Looking at the search terms the characteristics of the workforce has been explored in terms of the kind of professionals in the team. Foreign medical graduates have been mentioned. I think foreign graduates or better termed overseas graduates for all disciplines is significant, not just medical graduates.	Exploded MeSH terms were used in the search strategy testing for several of the databases which is why 'foreign medical graduates' were included. This was not a selected keyword and as such we had no control over its inclusion.
The other aspect in the composition of the workforce which would be notable to explore is permanent versus locum/bank staff. Does this impact on incidents would be significant. Bank/locum, permanent staff could be factored into the search words for workforce.	We agree that bank and locum staff should be in an important consideration in this review.<br>As such, we used the MeSH headings for workforce and healthcare workforce, which includes 'employment status'. We did not use bank or locum as a separate

	keyword search, as results still included these terms without limiting the search. We considered these as part of their 'characteristics' which we chose not to do a search string for and instead will use an inductive approach at extraction.
It is an important area of study and as the authors put forward, literature is limited. The review findings should be interesting and have an impact on workforce planning on dissemination.	Thank you to the reviewer for taking the time to review our manuscript and for their considered comments.
Reviewer 2	Response
Thank you for considering BMJ open for this very interesting systematic integrative review protocol. I appreciate the work involved in making the paper ready for peer review.<br><br>In my view the topic is very suitable for the journal and of an important subject. Exploring clinical staff/team characteristics within mental health hospitals and how these influence incidents of safety on wards is certainly an underexplored topic.<br><br>On the whole, I found the paper very well written, informative and accessible. In spirit of developing the paper, I have made suggestions below about how areas could be edited. By all means if you disagree, please note these disagreements in the response letter.	Thank you to the reviewer for taking the time to read our manuscript and for their reflections.<br>We have incorporated the suggested edits into the manuscript to strengthen the paper.
Introduction.<br>Line 8 Page 3: I was left wondering somewhat how safety incidents were contextualised as well as the clarity of the introduction section. Early on in the paper safety incidents is more defined by stating 'unintended and harmful consequences of clinical care'. Would it be possible please to use this 'definition' in the introduction? For example (I changed some wording which in my view aided clarity).<br>A neglected area of patient safety research is how the characteristics of	Thank you for this suggestion, we have edited the introduction of the abstract to reflect this.

mental health staff and teams may influence incidents, specifically, through unintended and harmful consequences of clinical care. Despite this, there is need to consider safety on mental health wards, for example the role of the staff team in containment and conflict. This review aims to explore the question, "How do staff and team characteristics relate to incidents in adult inpatient mental health settings?"	
Background.<br>Line 31 Page 4: There have been suggestions for relevant factors to consider in relation to safety on mental health wards, for example the role of the staff team in containment and conflict (10). I think 'to be considered' was meant to keep in the past tense?	This has now been edited (page 3, paragraph 2).
Line 45 Page 4: Perhaps add the term safety to 'incidents (12)'.	For consistency in language, 'safety incidents' is used throughout the manuscript.
Line 3 Page 5: I wonder if this sentence could be slightly adapted to 'Synthesising the literature across a range of staff and team characteristics, and their impact on a variety of safety incidents is therefore needed to progress safety research within inpatient mental health care'.	Edited to reflect comment (page 4, paragraph 1).
Line 13 page 5. In no way do I recommend the referencing of the following paper, especially given that I was one of the authors. But I would like to point out that there have been recent developments in relational care to aid patient feeling safe (in this case with their recovery) within acute inpatient settings. This, in turn makes me wonder a little about the accuracy of the following point: - 'There appears to be a lack of empirical evidence from mental health wards exploring patient safety in these settings compared to general healthcare settings.' If you wish to know more please see Deering, K., Wagstaff, C., Kyle, R.G., Bermingham, I., Williams, J. and Pawson, C., 2025. Safety for Recovery During	We appreciate the reflections of the reviewer. We have edited the sentence to recognise the growing body of literature (page 4, paragraph 2). However, we retain that patient safety literature as a collective in mental health settings is lacking compared to general healthcare.

Admission to Acute Mental Health Hospitals: A Constructivist Grounded Theory Study to Support Safety-Focused Recovery-Oriented Care in the Context of Risk Management Constraints. International Journal of Mental Health Nursing, 34(4), p.e70104. – thank you.	
Line 23 Page 5: Perhaps change staff error to staff errors or staff making errors? This is so it is clear that this refers to staff actions, not for example error in skills mix e.g. staffing error. Though of course this could be an issue.	Edited to reflect reviewer's comment (page 4, paragraph 2).
Line 28 Page 5. Perhaps a little pentadic on my part but 'allow' can regard permission while enable is for more causal to the methodological approach. E.g. will enable a wide range...	Edited to reflect reviewer's comment (page 4, paragraph 3).
Aim<br>Line 56 page 5. The question is slightly different as missing the term safety e.g. safety incidents. If OK with you when referring to the question just use the title of the paper - How do staff and team characteristics relate to ward safety incidents in adult inpatient mental health settings? Also, I was left wondering if the focus was on acute inpatient settings or all types of mental health inpatient settings. It might help therefore to clarify this also in the aim. This also could mean adapting to title of the paper to include acute, if seen reasonable to do so.	Edited to reflect reviewer comment (page 5, paragraph 2). The review is not limited to acute settings, as such we have not added this to the review question or the title.
Eligibility<br>I appreciate in the research terms at the end of the paper you are looking at particular dates but this is not clear in this section. Please add date range and rationale for searching the papers. Apologies if this is in this section but I could not see it. I also wonder if there might be a rationale provided for a mixed studies approach to the review? So, for example to develop a comprehensive picture around the impact of staff/team characteristics through observational and/or experiential studies.	The clarification has been added to the 'eligibility criteria' section on page 6.

Line 57 page 6: Regarding 'Literature including staff, patients, and families and carers of patients based in adult inpatient mental health settings', is this literature published also?	We have added 'published' to clarify (page 6, bullet point 2)
Information sources<br>Line 39 Page 7. It might be beneficial to provide a brief rationale for searching the identified databases in terms of the aim of the review.	A brief rationale has now been added (page 7, paragraph 1).
Search strategy<br>Line 52 Page 7: I wonder if the search should include the search term "characteristics" and another search without, as without investigating the term, this too might result in overlooking papers? Moreover, as I understand it, the term was utilised to understand the usefulness of conducting a meta-analysis later in the paper. This might leave readers wondering why the search strategy mentioned here was not utilised for understanding the need for a meta-synthesis approach.	Initial searches were conducted using "characteristics" and a list of those we were aware of from other literature to test what kind of results it would yield, and test against known papers. This approach did not add useful additional papers and when analysed in conjunction with other terms, limited the results and prevented characteristics which we had not identified from being included. As such an inductive approach was decided upon which has yielded much higher, relevant results and allowed for various definitions and language for characteristics to be identified.
Selection of sources of evidence<br>Line 10 page 8. I found this paragraph using a few incorrect terms and syntax. Please see a proposed edit of the paragraph as guidance. The changes are highlighted using [ ].<br>Results from each database will be exported into the [reference manager] software Rayyan where duplicate entries will be removed. Study selection will consist of two stages with the first [involving the] screening of titles and abstracts. This will be shared between two reviewers (KG and BG) independently screening 50% of the titles and abstracts each. Each reviewer will screen 10% of the other reviewers share to check agreement. Reviewers will meet [following] initial screening [deleted] [in which] disagreements will be discussed and resolved. If an agreement is not reached, the opinion of a third	Edited to reflect reviewer's comments (page 8, paragraph 1).

independent reviewer will be sought (SK). In the second stage, full text screening will be carried out independently by KG and BG and checked with the research team to ensure agreement. Each of these stages and the processes involved will be [consolidated into] a PRISMA flow diagram.	
Data charting process<br>Line 36 Page 8: Following inductive approach it might help the readers to understand its meaning, I suggest the following ...will follow an inductive approach (involving exploring the data to understand patterns), as outlined in guidance for integrative...	Edited to reflect reviewer's comment page 8, paragraph 2).
Quality appraisal<br>Line 52 Page 8: I am wondering if there should also be a mention about how exploring the quality of research can impact on the validity/trustworthiness etc of findings in review, and that such analysis will inform recommendations in the review to inform the conducting of future research on the topic.	Thank you for this reflection. We have added this consideration (page 8, paragraph 3).
Strategy for data synthesis<br>Line 5 Page 9: Perhaps write - which is large and [not but] heterogeneous.	Edited to reflect reviewer comment (page 8, paragraph 4).
Line 20 Page 9: I wonder if a meta-analysis was also not prudent due to different quantative study designs making it difficult to expand a sample size?	Thank you for this reflection. We agree and therefore have included this (page 9, paragraph 1).
Discussion<br>Line 32 Page 9: Regarding, 'short- and long-term', please remove the dash after short.	This has now been removed.
Line 55 Page 9 I think 'we have imposed' could be removed.	Edited to reflect reviewer's comment (page 9, paragraph 3).
Line 57 Page 9: A comma is needed after 'To mitigate the effects of this'.	Edited to reflect reviewer's comment (page 9, paragraph 3).
Line 14 Page 10: Regarding, 'in a practical way into operational...' I think in a practical way to inform operational – is perhaps better.	Edited to reflect reviewer's comment (page 10, paragraph 1).
Line 16 Page 10: Regarding, 'We anticipate this can make a significant contribution towards improved safety in	Edited to reflect reviewer's comment (page 10, paragraph 1).

inpatient mental health settings'. Again, I might be being a bit pedantic here but 'this' was not too clear to me. Using [ ], I wonder if the following is useful. We anticipate[, therefore, that the review findings will] make a significant contribution towards improved safety in inpatient mental health settings.	
Thank you for the opportunity to review this interesting paper and I wish you the very best with having it published.	Thank you again for your comments and suggestions for improvements to the paper.

VERSION 2 - REVIEW

Reviewer 1

Name Chakraborty, Nandini

Affiliation Leicestershire Partnership NHS Trust

Date 14-Feb-2026

COI

The authors have adequately addressed the queries and suggested changes to the protocol and manuscript.

Reviewer 2

Name Deering, Kris

Affiliation University of Exeter, Academy of Nursing

Date 11-Feb-2026

COI

Thank you for addressing my comments in the paper, I do not have further recommendations and wish you the best with having this protocol published.

Many thanks

Kris
